# Unlocking the potential of DNA-based tagging: current market solutions and expanding horizons

**DOI:** 10.1038/s41467-023-41728-2

**Published:** 2023-09-28

**Authors:** Adam Kuzdraliński, Marek Miśkiewicz, Hubert Szczerba, Wojciech Mazurczyk, Jeff Nivala, Bogdan Księżopolski

**Affiliations:** 1 Department of Cybersecurity and Cybereducation, Faculty of Information Technology, Polish-Japanese Academy of Information Technology, Warsaw, Mazowieckie 02-008 Poland; 2 Institute of Computer Science, University of Maria Curie-Skłodowska, Akademicka 9, 20-033 Lublin, Poland; 3 Department of Biotechnology, Microbiology and Human Nutrition, Faculty of Food Science and Biotechnology, University of Life Sciences in Lublin, 8 Skromna St., 20-704 Lublin, Poland; 4 Joint BioEnergy Institute, Emeryville, CA 94608 USA; 5 Biological Systems and Engineering Division, Lawrence Berkeley National Laboratory, Berkeley, CA 94720 USA; 6 Institute of Computer Science, Faculty of Electronics and Information Technology, Warsaw University of Technology, Warsaw, Nowowiejska 15/19, 00-665 Warsaw, Poland; 7 Parallelism and VLSI Group, Faculty of Mathematics and Computer Science, FernUniversität in Hagen, Universitätsstr. 1, 58097 Hagen, Germany; 8 Paul G. Allen School of Computer Science and Engineering, University of Washington, Seattle, WA USA; 9 Molecular Engineering and Sciences Institute, University of Washington, Seattle, WA USA

**Keywords:** Organizing materials with DNA, DNA computing

## Abstract

The commercialization of DNA tagging is a growing trend that demonstrates the increasing practicality of this novel approach. This interdisciplinary technology is based on the distinctive characteristics of DNA as a molecule that can remain stable in varying environmental conditions and store data following appropriate preparation. Moreover, newly developed technologies could simplify DNA synthesis and the encoding of data within DNA. The implementation of DNA tagging presents distinctive benefits in comparison to conventional labelling techniques, including universal product code (UPC) barcoding, radio-frequency identification (RFID), quick response (QR) codes, and Bluetooth technologies, by surmounting the limitations encountered by these systems. The discourse pertains to extant DNA-tagging mechanisms along with prospective implementations in a wide range of domains, including but not limited to art, the metaverse, forensics, wildlife monitoring, and the military. The potential of DNA labelling in various contexts underscores the importance of continued research and development in this rapidly evolving field.

## DNA-tagging technology

At present, several technologies are used to enable the tagging of physical objects, such as universal product code (UPC) barcoding, radio-frequency identification (RFID), near-field communication (NFC), quick response (QR) codes, and Bluetooth technologies^1,2^. While these technologies have many advantages, such as simplicity of application and the ability to automate the tagging and reading processes, they also have drawbacks. For instance, they cannot be applied to very small, flexible, numerous and physically changing objects or when the tag is to be hidden^2,3^. DNA-based tagging systems avoid these limitations.

DNA is a polymer that was found to be the primary source of data for living cells regarding their structure and function. Due to its high density, DNA is a potentially massive storage medium. For instance, assuming that data are kept in ssDNA (single-stranded DNA) molecules with two bits per nucleotide (nt; for example, A = 00, T = 01, C = 10, and G = 11), we can store 455 exabytes of data in 1 g of DNA^4^. Archaeological studies have also determined that DNA information is very stable, since it is possible to read the sequence of mitochondrial DNA from samples that are 300,000 years old^5^.

DNA taggant design is the crucial step in tagging technology, which involves determining (i) the type and size (in bits) of information contained in DNA tags, (ii) the information coding method, (iii) the method of storing information in a tag, and (iv) the method of reading or extracting the data.

Notably, the concept of a DNA-tagging system is not a recent proposal as it was developed beginning in the 1980s. The early approaches to the technology were addressed in patent WO1987006383^6^, published in 1986, which outlines an invention that encompasses a distinctive labelling procedure. The method entails the application of a preselected macromolecular compound (referred to as the “signal compound”) to label the item or substance. The aforementioned signal compound possesses the ability to bind with a complementary compound, thereby indicating the presence of the initial compound and verifying the genuineness of the item or substance. The authors posit that the “signal compound” could potentially be DNA. Shortly afterwards, Cetus Corporation, a renowned entity recognized for its breakthroughs in PCR technology, filed a patent application under the reference WO1990014441^7^, presenting a new technique for monitoring the presence of a particular substance. The method involves the attachment of a nucleic acid to the substance, the collection of the substance, and the detection of the aforementioned nucleic acid. In the years that followed, patent applications were filed for using nucleic acids to tag various physical items, including liquids^8–13^. However, due to the limitations of this technology, work on its improvement is still ongoing.

A major challenge in DNA tagging is the instability of nucleic acids caused by external factors, including temperature, humidity, UV light, molecular oxygen, nucleases, or metal ions^2,14^. Therefore, one of the primary steps in building DNA-tagging systems is protecting DNA against degrading agents by creating a physical barrier between the DNA and the environment. For this purpose, silica, polymers, and gels are frequently used^15,16^.

Another obstacle is decoding the DNA tag. PCR (polymerase chain reaction) or its variants are methods for determining the presence of a specific tag. Berk et al.^17^ described a method for reading DNA security tags either by sight or with a smartphone. In this approach, sequence-specific strand-displacement assays, placed on a paper ticket, are arranged in a specific way, allowing a fluorescent spot pattern to appear after exposure to an appropriate taggant. The validating pattern can be generated by a taggant only if it contains the appropriate DNA sequence(s).

Ongoing research has also demonstrated that the implementation of CRISPR-based readout techniques, including SHERLOCK, exhibits considerable potential in the area of DNA tag identification and decoding^18^. The utilization of the SHERLOCK system, which combines recombinase polymerase amplification (RPA) with a Cas13a-based nucleic acid detection assay, has been implemented for the identification of tags present within synthetic spores referred to as barcoded microbial spores (BMS). The solution has been tested on different surfaces in simulated real-world environments, demonstrating its potential for wide-ranging applications in determining the origins of objects and tracking their movement^19^.

DNA sequencing represents an alternative approach, enabling either verification of the DNA tag’s identity or decoding of the tag’s data^3^. Although the application of these technologies still requires a well-equipped laboratory and complex procedures, these obstacles can be surmounted by rapidly developing portable technologies such as SmidgION^20^, developed by Oxford Nanopore Technologies (ONT).

**Fig. 1:**
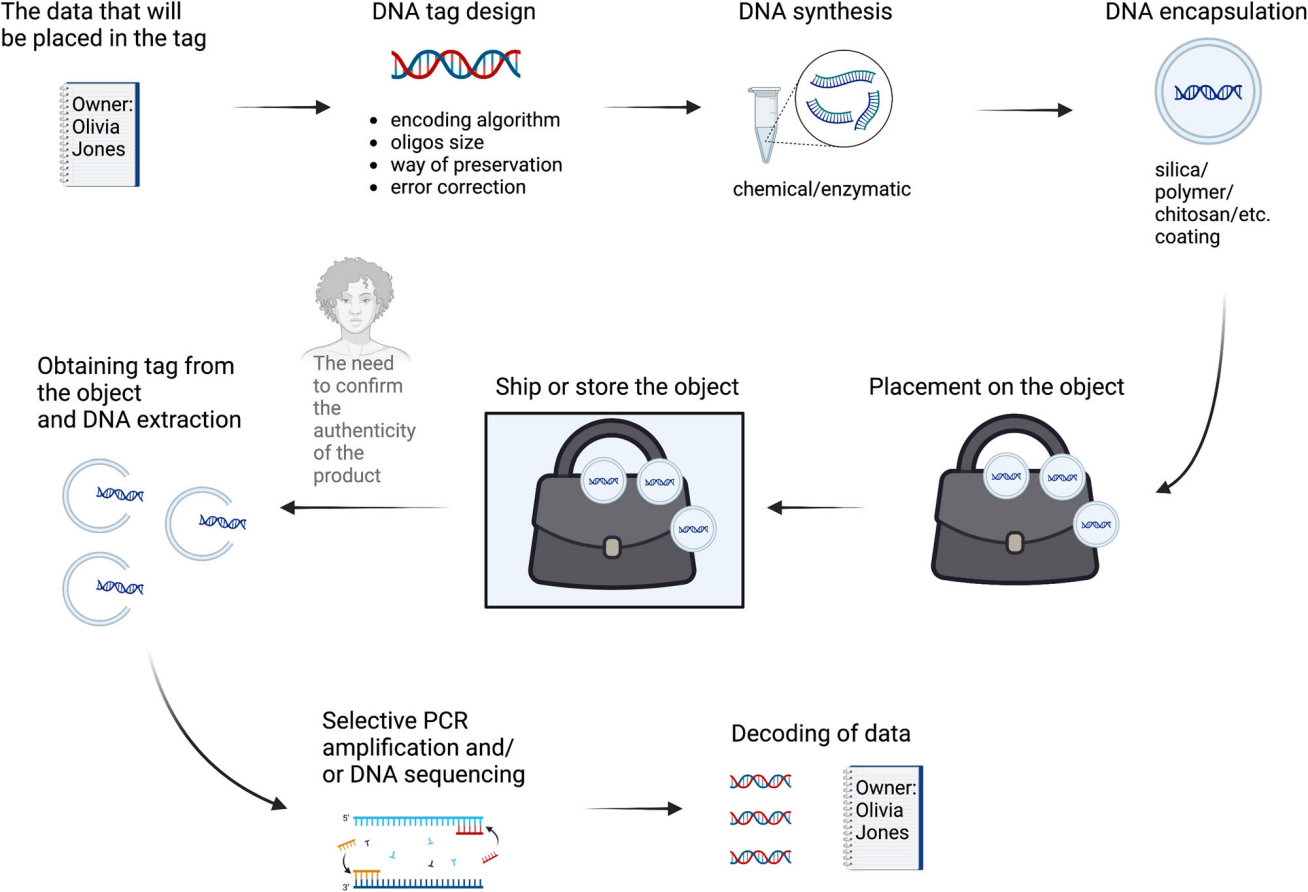
The crucial steps in a DNA-based tagging system. The procedure may begin with application to a physical object. Most tangible items are suitable for DNA tagging, although the duration of DNA viability on their surface may depend on the item’s properties. The DNA tag has the potential to be integrated into the object’s structure, for instance, as a constituent of a polymer or pigment. The initial stage in verifying the presence of the tag on the tangible item is the extraction of DNA. The exact location of the tag on the object may be masked. However, it could also be marked by a particular label or dye (e.g., visible under UV light). The subsequent stage in the overall process involves tag reading, which can be executed through either DNA amplification in conjunction with DNA sequencing or DNA sequencing alone. One of the fundamental techniques involves the amplification of DNA strands through conventional DNA amplification, applying two primers, followed by sequencing. Ultimately, the sequence derived from the tag can be decoded and transformed into information.

Its core concept is explained in Fig. 1.

## Existing DNA-tagging systems

Porcupine, developed in a partnership between the University of Washington and Microsoft (USA), is a complete DNA-tagging system described in the scientific literature^3^. The specifics of its design and functionality are therefore known. The molecular bits (molbits) used in this portable end-to-end molecular tagging system, which does not require access to specialized laboratories or equipment, incorporate highly separable nanopore signals to facilitate subsequent readout. In the molecular tag mixture, the presence or absence of particular molbits is represented by 1 and 0, respectively. They categorize molecular tags directly from the raw nanopore signal using a portable nanopore device provided, for instance, by ONT third-generation sequencers, thereby omitting the base calling process of converting DNA back to sequence at the readout stage.

Other concepts related to the utilization of DNA tags are expounded in the scientific literature. The development of DNA ink involves the combination of synthetic DNA with printing inks^21^. Another approach involved the combination of double-stranded DNA with a pigment that is sensitive to infrared light and exhibits fluorescence when exposed to ultraviolet light. In another study, DNA molecular tags were integrated into lactose tablets or pharmaceutical-grade printing ink as a potential anti-counterfeiting monitor in the pharmaceutical industry^22,23^. The study conducted by Puddu et al.^24^ involved the encapsulation of DNA within magnetic particles that are both heat-resistant and inert, demonstrating potential for food tracing.

Apart from the scientific investigation of DNA tagging, commercial enterprises offer DNA-oriented technologies for the purpose of labelling tangible objects. Although many of these organizations do not divulge the precise details of their inventions, it is possible to uncover certain aspects to some degree^25–27^.

SigNature DNA is a botanical DNA-tagging technique that has been developed by Applied DNA Sciences. The detection of DNA taggants is accomplished by PCR with specific primers and capillary electrophoresis (CE). SigNature DNA taggants have the capability to be incorporated into a range of marking systems, including but not limited to RFID devices, labels, serial numbers, and holograms^22,25,28–31^.

The Haelixa company provides a DNA taggant that is enclosed in silica capsules with a diameter of ~100 nm. This enables the protection of delicate DNA and its attachment to tangible goods^32,33^. In addition, this company offers a liquid solution that contains DNA. The identification of a DNA tag is accomplished through the utilization of PCR or modifications^34,35^.

Next, SelectaDNA (the brand of Selectamark Security Systems) developed a two-step system whereby DNA serves as an alternative authentication solution if the primary method is unavailable. In scenarios of property theft recovery, law enforcement officials utilize a UV lamp to identify adhesive markings and a magnifying glass to locate microdots. This enables them to extract the registration code and contact information, which can then be used to retrieve the owner’s details from the service provider’s database. If microdots are not present, a laboratory analyses the unique DNA composition to identify the owner. The genetic material’s structure involves the integration of two discrete synthetic nucleotide oligomers, each of which encompasses a binding sequence for a primer and an identifier sequence. The tag appears to be read by DNA sequencing^26,36,37^.

CypherMark technology (TraceTag) is designed with a pair of primer sequences, also known as keys, and the detection of tags is executed through the utilization of qPCR (quantitative PCR)^15^. A suggested utilization entails the dissemination of a composite comprising the nucleic acid marker and marking fluid onto objects, such as currency notes. TraceTag offers a range of DNA inks suitable for different applications^27,38^.

Holoptica offers DNA SmartMarks, which combines distinctive DNA segments, additional biological markers, and visual indications. Optical indicators utilize a laser scanner that is calibrated to generate spectral and temporal signals to electronically or visually confirm the existence and legitimacy of a mark. The DNA component is a molecule consisting of 100 base pairs that can be incorporated into an inkjet cartridge^39,40^. The patent filed by Holoptica delineates a technological innovation that involves a base layer of metallized foil with an integrated QR code. This is further augmented by the application of moulded layers of synthetic DNA, nanoparticles and, finally, a protective transparent film^41^.

DNA Technologies has developed a technology that incorporates DNA-laced ink for tagging and safeguarding valuable products, brands, and intellectual property. The technology, known as “DNA Matrix™” involves adding DNA taggants with unique photoluminescent properties, including activation frequency and fluorescence fading patterns, to printing inks. These characteristics render each mark machine-readable, enabling differentiation between genuine and counterfeit items through specialized scanners. Originating as an art authentication system, DNA Technologies has since expanded its applications across various industries, including pharmaceuticals^42,43^.

Tagsmart introduced Smart DNA Tags in 2016. The company specializes in the authentication and security of artworks. The utilization of synthetic DNA tags for identification purposes is facilitated by linking them to an online platform. Each individual tag possesses a distinct reference number that is associated with the artwork’s secure certificate of authenticity and its corresponding digital version. The aforementioned technology has also been applied to the manufacturing of premium books^44,45^.

The DNA Guardian system employs technologies that are subject to limited information availability. It involves the use of a marking agent that contains nucleic acid, along with a UV-detectable stain^46,47^.

Aanika Biosciences has been developing microbial tags that can be used as biological barcodes for various food items, such as grains, fruit, and vegetables. The aforementioned tags employ bacterial spores. However, many technical details about genetically engineered bacterial spores remain undisclosed. The use of genetically modified organisms (GMOs) in Aanika Biosciences’ technology also raises concerns regarding the potential release of these organisms into the environment, and their presence in food may elicit distress among consumers^48–50^.

Table 1 presents all the companies described in the text, selected features of the technologies they use, and the markets that are described in terms of the application of individual DNA-tagging technologies.

**Table 1:** Companies that provide or create technological solutions for labeling tangible items through the application of DNA

Company name^a^, country of origin and launch date	Main features of the technology	Selected markets	References
Applied DNA Sciences, United States, 1983	Botanical DNA fragments, detection by PCR and CE, an encapsulation system	Product authentication, supply chain traceability, brand protection, anti-counterfeiting, textiles, pharmaceuticals, etc.	^12,15,18–21^
Haelixa, Switzerland, 2016	Synthetic DNA tags, detection by PCR, DNA enclosed in silica	Product authentication, supply chain traceability, intellectual property protection, etc.	^22–25^
Selectamark Security Systems (SelectaDNA), United Kingdom, 1986	Laboratory analysis of DNA for owner identification if microdots are absent (DNA serves as an alternative authentication solution)	Asset protection and recovery, securing high-value items, art and jewelry authentication, IT equipment and vehicle security, forensic applications, theft prevention and deterrence, etc.	^16,26,27^
TraceTag (CypherMark), United Kingdom / Norway, 2001	Synthetic DNA with unique primers, authorized access to primer sequences, detection using qPCR	Brand safeguarding, industrial applications, cash security, security of documentation, oil and fuel tracking, anti-counterfeiting measures, etc.	^7,17,28^
Holoptica, United States, 2012	Synthetics DNA tags (100 nucleotides), integration with inkjet cartridges	Artwork, documents and assets protection, verifying product authenticity, food tracking, etc.	^29–31^
DNA Technology, United States, 1993	DNA-laced ink, combination of DNA synthetic segments and optical taggants	Memorabilia and collectibles, limited edition artwork, pharmaceuticals, apparel and luxury goods, health and beauty industry, etc.	^32,33^
Tagsmart, United Kingdom, 2015	Synthetic DNA tags, secure Certificate of Authenticity	Artwork, securing collectibles, verifying paper documents, book manufacturing, etc.	^34,35^
DNA Guardian, Australia, 2007	UV-detectable stain, detection using pyrosequencing	Asset marking, crime prevention, artwork protection, theft deterrence, etc.	^36,37^
Aanika Biosciences, United States, 2018	Genetically modified *Bacillus subtilis* as an encapsulation system for DNA tag	Agriculture and food production, textiles, etc.	^38–40^

^a^Brand or technology name.

A considerable fraction of the abovementioned companies employ short DNA strands, with a maximum length of a few hundred nucleotides, which are recognized through conventional PCR, qPCR, or occasionally DNA sequencing. The lack of information pertaining to the implementation of sophisticated technologies, such as the storage of larger quantities of data inside longer DNA molecules, is evident. This could be supported by the increased probability of the perseverance of molecules with shorter lengths in diverse conditions^51^. Nevertheless, a diverse array of mechanisms are employed to protect DNA, spanning from silica microbeads to bacterial cells. In particular cases, DNA is employed as an additional means of verification, rather than serving as the principal method of recognizing an object. SelectaDNA, for instance, is a company that utilizes microdots as its principal taggant technology while simultaneously incorporating DNA as an extra layer of security^26,36,37^. The main justification for this is that the validation of a DNA marker’s presence within a laboratory increases the expense of the procedure and prolongs the timeframe for the delivery of results.

Notwithstanding the potential advantages of DNA-tagging technologies, it is crucial to consider their drawbacks and limitations. The current use of laboratory analysis of samples to identify DNA tags may limit the ability of these technologies to scale up. On the other hand, PCR’s great sensitivity allows the use of minimal quantities of DNA tags, since the tag could theoretically be detected from as little as one DNA molecule^52^. However, high sensitivity may also lead to false positives or increase susceptibility to contamination. Finally, the claim that DNA tags cannot be copied, which is supported by numerous companies, may be contested by the prospective use of next-generation sequencing methods that do not require DNA fragment amplification before sequencing^53^.

## Future prospects

The characteristics of DNA, including its capacity for extended preservation, resilience, and amplification, make it a potent tool for the identification and tracking of physical objects. While tangible item tagging methods have been utilized in commercial settings for many years, DNA-based tagging holds promise as a next-generation approach to the advancement of such technologies. Despite the presence of numerous DNA-tagging service providers in the market, it seems that the exploitation of this technology is still limited compared to the widespread utilization of QR, RFID, and UPC technologies. Furthermore, novel applications for DNA-tagging technology are expected to be limited in the future, and its development will be predominantly propelled by technological advancements, particularly in synthesis and tag encoding, which could make existing applications more practical to address by molecular tagging.

Currently, significant improvements are being made in the field of sequencing technology, as exemplified by the arrival of third-generation sequencers, such as those manufactured by ONT. Notably, these sequencers are anticipated to be compatible with mobile devices, which could potentially facilitate the application of these sequencers as instruments for DNA tag reading without requiring a laboratory environment^54^.

The labelling of DNA exhibits significant potential for application across diverse market segments. Many of these technologies are currently available, but mostly with a limited commercial starting point. Several startups are seeking to create and implement these technologies and their corresponding applications^49,55^. Possible uses may involve the verification and monitoring of the ownership of high-value artwork or its packaging, facilitating reliable and straightforward authentication. The monitoring and tracing of pharmaceutical products from their production site to their point of purchase could be deemed a crucial application, as it guarantees the authenticity and security of declared products.

This new technology has the potential to become a widespread system of authentication for premium luxury goods, such as high-end watches and designer handbags. Furthermore, the implementation of DNA tagging could potentially serve as a dependable method for authenticating human-associated documentation, such as passports and driver’s licences. The implementation of this technology within administration scenarios could render counterfeiting of these documents highly challenging, if not impossible.

The potential to generate synthetic DNA strands and subsequently encode information in the form of a nucleotide sequence presents opportunities not only for the creation of identification tags but also for the broadening of their prospective applications in the field of cryptography. Numerous studies have demonstrated the potential of utilizing the characteristics and mechanisms of nucleic acids to construct cryptographic systems of substantial complexity^56^. The implementation of the aforementioned ideas is exemplified by Cui et al.^57,58^. The study proposed by the authors incorporated encoded data into microdots of DNA fragments, which were subsequently applied to paper via spray deposition. This method rendered the information imperceptible to the naked eye and presented a formidable challenge to any individual attempting to decode the encrypted content. This technology has the potential for broader application in the fields of secure data storage and sensitive communications in sectors such as defence, intelligence, or healthcare data protection.

DNA tagging turns out to be essential in connecting material objects or specific aspects of them to their tangible representations^55^. Employing this technology, it becomes plausible to devise analogues to nonfungible tokens (NFT) for concrete items. Immutable and tamper-proof DNA tags could lay the groundwork for constructing systems that facilitate the application of principles such as nonrepudiation, authenticity, and accountability to real-world objects. Such principles, until now, have been predominantly associated with the digital domain.

The application of DNA to the identification and surveillance of tangible objects can be expanded to include the connection with corresponding virtual entities or items within the realm of the metaverse. A tangible entity present in the physical realm can be linked to its digital representation through the application of a unique DNA tag.

DNA tags have the potential to be attached to a diverse range of objects, such as clothes, weapons, and various other items. There is a possibility that DNA tagging of clothes, such as that offered by companies such as Applied DNA Science and DNA Security Solutions, will eventually become a widespread practice^22,25,56^. The footwear and clothing industry has experienced significant economic losses due to the proliferation of counterfeit products^59^. This implies that the currently implemented preventive systems for such issues lack effectiveness. Another potential application involves the implementation of tagging technology on weaponry, including bullets, to ascertain the origin of weapons used in military conflicts. Additionally, the method has the potential to monitor the displacement of armed forces and resources in times of armed conflict. As proposed by Aanika Biosciences, DNA tagging could become a widespread, mainstream method to monitor the movement of agricultural products along the entire supply chain. Additional potential applications include tracing the provenance of valuable and scarce minerals, such as diamonds, which are listed in the description of the applicability of the Haelixa tagging system^60^. The widespread utilization of DNA-tagging technology could potentially become a significant component in tracking diamonds from their mining origins to the importation stage. This system could become a component of larger initiatives such as the Kimberley Process Certificate, which certifies that raw diamonds originate from conflict-free regions^61^. This becomes particularly significant as diamonds are used by rebel movements or partners to fund their military actions.

Another possible application might be monitoring endangered animals by following marked individuals or cohorts, for instance, via spray-marking. The use of DNA tags on the various components, products, and wastes of industrial processes has the potential to enhance efficiency, minimize waste, and ensure adherence to regulatory standards.

**Fig. 2:**
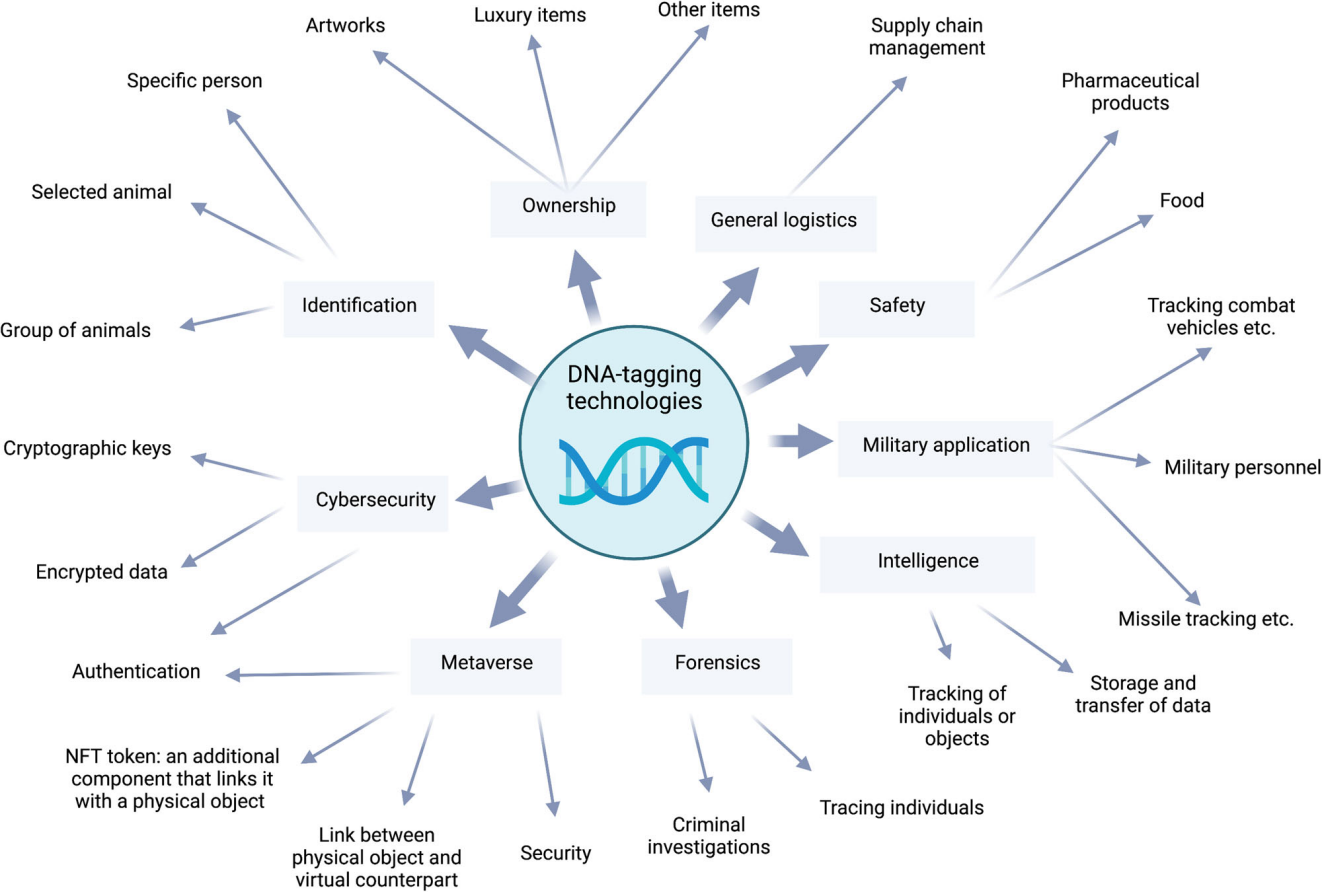
Examples of applications of DNA-tagging technology. The process of tagging items with DNA presents a diverse range of potential applications. The “DNA-of-things” concept suggests that the incorporation of DNA into physical objects offers the potential to imbue these objects with a unique characteristic that enables their identification in a manner akin to that of living organisms^62^. The DNA polymer utilized may possess unique characteristics and could be linked with either the individual or the organization that possesses the item, such as artistic creations and inventions. Furthermore, the utilization of DNA tagging presents the possibility of establishing a connection between the concept of the metaverse and reality, particularly in scenarios that require validation of the connection between a tangible object and its virtual counterpart, or vice versa. The DNA tag exhibits the potential to serve as a supplementary feature to NFTs for tangible objects, validating the authenticity of the chosen object. The use of DNA tagging may demonstrate a correlation between a specific person or animal and a particular location. In cases where criminals often utilize cunning tactics to mask their identity, the application of a DNA tag spray turns out to be highly advantageous for the field of forensic science. Comparably, DNA tagging can be used to label animals without the need for animal tracking devices, which can interfere with the animal’s natural behaviour or become damaged or misplaced. By combining DNA-tagging characteristics and the potential for data storage, which may also be secured using currently available cryptographic techniques, DNA can also be used for military and intelligence objectives. Applications for DNA labelling in cybersecurity can function as a deterrent to the emerging threat posed by quantum computers, whose computational power will render many currently employed encryption schemes worthless. One potential solution involves integrating encryption methodologies with DNA tagging, thereby enabling a linkage to the user’s genetic material. Deciphering such a key would pose a significant challenge. The use of DNA labelling in food and pharmaceutical production is supported by a strong safety argument. When tagged, such products will become impossible or extremely difficult to counterfeit. Estimates suggest that 10.5% of the worldwide medicine supply is counterfeit, including 13.6% in developing countries. The sale of counterfeit medications rose from $75 billion in 2009 to $600 billion in 2018. The World Health Organization (WHO) attributes 0.2 million of the 1 million annual malaria-related deaths to counterfeit anti-malarial drugs. Amidst the COVID-19 pandemic, a recent report asserts that the illicit drug market experienced a surge of over 400% towards the end of 2021^63–65^.

Figure 2 summarizes the main directions and areas of application for the possible use of DNA tagging in the future.

The field of DNA tagging is an interdisciplinary area that involves cooperation among experts from various domains, including chemistry, biotechnology, computer science, materials science, biology, and physics, to address significant challenges. Ongoing technological advances may lead to a rise in the popularity of DNA tagging in the market, potentially revolutionizing various industries.

## Data Availability

We do not analyse or generate any datasets.
